# Validation of absolute dose calculation of a single ^125^I-seed for ophthalmic brachytherapy within distance range 1–15 mm using a diode detector

**DOI:** 10.2340/1651-226X.2026.45688

**Published:** 2026-05-27

**Authors:** Anna Rintala, Vappu Reijonen, Assi Valve, Mikko Tenhunen

**Affiliations:** a Helsinki University Hospital, Comprehensive Cancer Center, Helsinki, Finland; b Department of Physics, MATRENA, University of Helsinki, Helsinki, Finland

**Keywords:** Iodine-125, diode, dosimetry, brachytherapy

## Abstract

**Background and purpose:**

Intraocular tumors can be treated with brachytherapy. Dosimetry of ophthalmic applicators is challenging due to short distance ranges and steep dose gradients. Measurements are sensitive to the structure of the dosimeter, and correction methods specific to ^125^I-source are needed for validation measurements of dose distribution.

**Material and methods:**

We measured dose profiles of I25.S16 seed using diode detector in a water phantom at 2 and 5 mm above surface of acrylic (PMMA) and gold backing. Volume averaging effect was corrected by the ratio of dose calculated with AAPM TG-43 formalism at the center point and the dose average within the detector element area. The angular sensitivity was measured at a constant radius around the seed. The effect of gold backing was evaluated from measurements with and without gold slab behind the seed. Measurements were normalized to calculated value at the distance of 10 mm from the seed using the air-kerma rate measured with a calibrated well chamber.

**Results:**

1D/3D gamma comparison pass rates 97% with criteria 0.3 mm/3% and 60% with 0.5 mm/5% were reached for a seed attached to PMMA and gold, respectively, in the range ± 10 mm.

**Interpretation:**

TG-43 calculated single seed distributions in PMMA were equivalent to measurements corrected for angular sensitivity and detector area. Gold-to-water ratio corrected calculations were not similarly comparable, and simple one-dimensional correction method is not accurate enough laterally. Diode dosimetry with these correction methods can be used for quality assurance and validation of clinical dose calculation of ophthalmic applicators.

## Introduction

Intraocular tumors, such as uveal melanoma, can be treated with ophthalmic brachytherapy. There are many different designs of ophthalmic applicators, in terms of shape and composition of the plaque, radionuclide source employed and how they are affixed to the plaques. One of the most widely used configurations consists of low-energy photon-emitting seeds assembled to the inner surface of a cap made of gold alloy [1, 2].

Dosimetry of brachytherapy sealed sources has been based on the American Association of Physicists in Medicine (AAPM) Task Group 43 (TG-43) dose calculation formalism [3], though there is an aim to transition toward model-based calculation algorithms [4]. Monte Carlo (MC) calculations are the predominant method for determining values for source specific functions used in the TG-43 formalism [5]. One essential limitation of TG-43 is that it assumes full scattering conditions in water [1]. It considers neither tissue heterogeneities nor, more importantly, the backing of ophthalmic applicator. The main effect of the backing is decrease in dose due to the lack of backscattering behind the applicator. It is recommended that for accurate patient dose calculation the effect of backing is taken into account [6]. This is even more important in the case of collimating plaques, where the lips of plaque or single seed slots are used to limit the distribution [2].

As a part of the quality assurance of ophthalmic brachy-therapy, calculated dose distribution of an applicator should be validated [1]. However, it is challenging due to the short distance ranges and steep dose gradients from the sources. Small errors in detector positioning translate into large errors in dose deduced from the detector signal. Many types of dosimeters have been used, and detector response artifacts should be minimized or accurately accounted for by appropriate correction coefficients.

Silicon diode detectors have been used for relative dose distribution measurements of ^125^Iodine (^125^I) sources, for example, in single seed characterization study [7], investigations of the effect of backing materials [8, 9] and verification of dose distribution of multiple seeds in applicators [10]. The advantages of diodes are small effective size, high current per unit absorbed-dose rate and the possibility of scanning measurements with almost immediate readout in a water phantom system. The response of diodes is dependent on photon energy, but for ^125^I the energy spectrum changes very little with distance [11].

Correction methods verified with single seed measurements are a prerequisite for multiple seed dose distribution measurements because with TG-43 formalism, eye plaque distribution is generated as superposition of single source contributions. Our aim was to develop and validate a measurement set up with a diode detector for dose distribution measurements of custom-made eye plaques [12] used in our clinic. At first, the performance of correction methods for diode signal was demonstrated in water equivalent environment down to very short distances (1 mm) from the ^125^I-seed center. Finally, the methods were tested against measurements with an applicator surrogate, together with a simple one-dimensional correction for the effect of gold backing applied to calculation.

## Material and methods

### Measurement setup

We investigated the properties of p-type Si-diode (1110000-1, Sun Nuclear U.S.) for measuring ^125^I sources. The diode detector has a 0.3 mm entrance window in front of the effective point, and the area of the detector element is a square with 2.3 mm diagonal and thickness of 50 µm.

**Figure 1:**
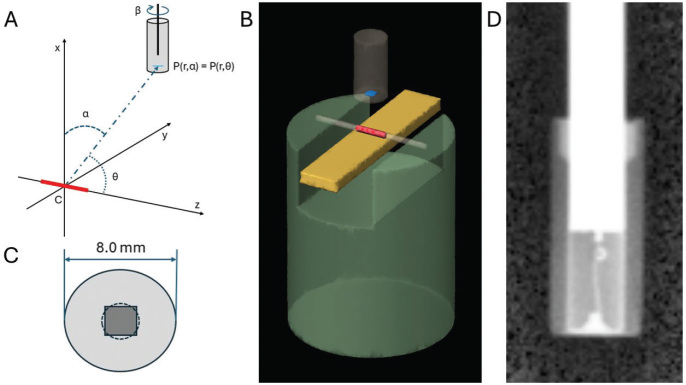
(A) Schematic illustration of the coordinate system of the measurements. The ^125^I seed (red) lies along the *z*-axis in the horizontal *zy*-plane. The angle *α* denotes the angle between line PC, where point P is the diode position and C is the center of the seed at the origo, and the vertical *x*-axis. The angle *θ* of the TG-43 protocol is the angle between the line PC and the *z*-axis. The angle *β* is the angle of rotation of the diode detector around its own vertical axis. (B) Simplified illustration of the PMMA holder used in the measurements. The diode detector (gray, effective area in blue) is moved with respect to the seed (red), which is positioned in a plastic catheter which is glued to the holder. The gold slab (yellow) can be positioned behind the seed. (C) The square detector element is approximated as a circle of the same area. (D) X-ray image of the diode detector.

All diode measurements were carried out in a MP3 water phantom (PTW Freiburg GmbH, Germany) whose detector positioning mechanism supports orthogonal movements with 0.1 mm steps. The coordinate system used in this study is depicted in Figure 1A, where *x*, *y* and *z* represent orthogonal coordinates of the point of interest (measurement) from the seed center and $r=\sqrt{x^{2}+y^{2}+z^{2}}$ is the distance from the seed center. α is the angle between *x*-axis and the measurement position. *θ* is the angle between *z*-axis, parallel to the linear source, and the measurement position. Variables *r* and *θ* follow the notation used in TG-43 formalism. Custom made PMMA holders (Figure 1B) were attached to the side of the water phantom. The PTW TANDEM electrometer was used for scanning measurements and standalone PTW UNIDOS Romeo electrometer for point measurements with diode bias voltage of 0 V.

The ^125^I sources that we used in this study are I25.S16 (Eckert & Ziegler Medical, Germany) seeds (physical length 4.5 mm and thickness 0.8 mm, active length 3.5 mm and thickness 0.59 mm). The air-kerma rate of each seed was determined with a calibrated reference-type well chamber (HDR 1000 Plus, single seed holder 70016 (Standard Imaging Inc., U.S.), calibration uncertainty 3.3%) and ambient conditions tracked appropriately [13]). The air-kerma rates of the seeds were between 0.098−0.169 μGy · m^2^ · min^–1^ at the time of the measurements.

We calculated dose rates *Ḋ*_TG−43_ (*r*,*θ*) with TG-43U1 formalism [3] using radial dose function *g*(*r*) fit and anisotropy function *F*(*r*, *θ*) values given in CLRPv2 database [5, 14]. Consensus value [3] was used for the dose-rate constant in water Λ = 1.012 cm^−2^ converting air-kerma to absorbed dose at the TG-43 reference point *r*_0_ = 1 cm and *θ*_0_ = 90°. The geometry function *G_L_* (*r*, *θ*) was calculated according to the 3.5 mm long linear source.

### Area averaging effect

The diode signal averaging effect within the sensitive area is modeled supposing constant sensitivity within the detector area. The dose gradient in the sensitive detector area is dependent on the detector distance *r*, direction from the source to detector *θ* and the angular position α of the detector with reference to the source (Figure 1A). The correction for dose averaging within the detector element *C*_avg_ (*r*, *θ*, α) was evaluated computationally from the ratio of dose rates calculated at the center point (*r*_c_, *θ*_c_) and the average within the area of detector element approximated as a circle (Figure 1C):


$$C_{avg}\left(r_{c},\;\theta_{c},\;\alpha_{c}\right)=\frac{{\dot{D}}_{TG-43}\left(r_{c},\;\theta_{c}\right)}{{\bar{\dot{D}}}_{TG-43}\left(r,\;\theta \right)}$$ (1)

The detector element is square-shaped, but its rotational position within encapsulation is unknown.

### Angular sensitivity

Angular sensitivity was evaluated from measurements at a constant radius of 10 mm around the seed axis (*θ* = 90°) where the dose rate is constant. The seed was positioned in a water filled plastic catheter (wall thickness < 0.3 mm). Charges Δ*Q* were integrated over time Δ*t* = 1 min at angular range -150°≤ *α* ≤ 150°, where α is the angle between vertical direction and detector position as in Figure 1A, at 10-degree intervals except 5-degree intervals where the change in the sensitivity is greater. Small corrections were made to the measured values due to the discrete steps of detector positioning mechanism resulting in deviations from the intended radius.

X-ray image of the inner structure of the diode is presented in Figure 1D. Angular sensitivity was measured at four rotational positions (*β*) around the detector axis at 45-degree intervals. All four curves were normalized to one at angle α = 0° mirrored through it and averaged to get the correction factor *C_ang_* (*α*), which is an inverse of sensitivity values:


$$C_{ang}\left(\;\alpha \right)=\frac{\bar{\Delta Q}(\alpha =0{}^{\circ})\cdot C_{avg}(r=10\text{ mm},\;\alpha =0{}^{\circ})}{\bar{\Delta Q}(\alpha ,\;\beta )\cdot C_{avg}(r=10\text{ mm},\;\alpha )}$$ (2)

For the correction of profile measurement values, linear interpolation between two nearest measured angular points was used. The angular sensitivity was assumed to be constant as a function of distance *r* or direction *θ* from the source.

### Effect of gold backing

The effect of the gold slab used in the profile measurements was evaluated from point measurements in the *x*-direction. Measurements were made with and without the 11 × 44 × 2 mm (width × length × thickness) 24K gold slab just under the seed positioned in a catheter (Figure 1B), and the gold values were divided with water values.

The value of gold-to-water dose ratios *C_gold_*(*r*), where *r* is the distance from the seed center, used to correct calculated dose values, were evaluated from an experimental fit function


$$C_{gold}\left(x\right)=a\cdot \exp\left(b\cdot x\right)+c.$$ (3)

Thus, the water-to-gold corrected calculated dose rates are:


$${\dot{D}}_{TG-43,gold}\left(r,\theta \right)=C_{gold}\left(r\right)\cdot {\dot{D}}_{TG-43}\left(r,\theta \right).$$ (4)

### Dose profile measurements

A single ^125^I seed was attached with silicone glue on the surface and in a slot, respectively, of both acrylic (PMMA) and a gold slab. The depth of the slot was the same as the radius of the seed (0.4 mm). Horizontal centering of the position of the diode detector with respect to the center of the seed was done with profile scans. In the vertical direction, position was determined from the surface of the slab taking the depth of the effective point of the diode into account. Depth doses (“xprof”) were measured from 1 to 25 mm above the surface and profiles in both short (“yprof”) and long (“zprof”) axis directions 2 and 5 mm above the slab surface at range ± 15 mm from the *x*-axis.

Dose distribution measurements ∆*Q*(*r*, *θ*) (integration time Δ*t* = 2 s) were normalized to correspond to dose rate in water for the I25.S16 seed that has air-kerma rate *K* of 1 mGy min^-1^ at the point *P*(*r*_0_ = 10 mm, *θ*_0_ = 0°) from the seed center based on the air-kerma rates corrected to the time of the measurement.

The reading of the diode was calibrated to average of corrected point measurements at *r*_0_ = 10 mm in water (seed in a catheter):


$$k_{calib}=\frac{\dot{K}(r_{0},\;\theta_{0})\cdot \Lambda }{{\bar{\Delta Q}}_{norm}\left(r_{0},\;\theta_{0}\right)\cdot C_{avg}\left(r_{0},\;\alpha =0\right)}.$$ (5)

The detector signal values were normalized with the calibration coefficient and corrected with the angular sensitivity and area averaging factors:


$${\dot{D}}_{meas}\left(r,\;\theta \right)=k_{calib}\cdot C_{ang}\left(\alpha \right)\cdot C_{avg}\left(r,\;\theta ,\;\alpha \right)\cdot \Delta Q_{norm}\left(r,\;\theta \right).$$ (6)

Measured and calculated dose profiles were compared with 1D/3D gamma (*γ*) index analysis with distance to agreement/dose difference (DTA/DD) criteria 0.2 mm/2%, 0.3 mm/3% and 0.5 mm/5%. Dose difference was normalized to the calculated value at the calibration point, that is, global normalization was used. The threshold of passing the gamma comparison was set to 90%. The DTA/DD criteria were chosen based on the expected performance limit of the dosimetric method – so that pass rates worse than 100% would indicate the achieved accuracy limit – and not according to the actual clinical needs of the ophthalmic brachytherapy treatment modality. However, considering the size scale of ocular structures, submillimeter criterion is relevant.

## Results

### Area averaging effect

**Figure 2:**
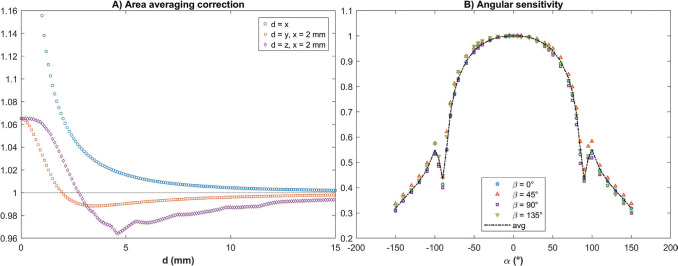
(A) Examples of area averaging within the detector element approximated as a circle along or parallel to the main axes *x*, *y* and *z*. (B) Angular sensitivity of the diode detector measured at four different rotational positions *β* around the detector’s own axis and the averaged sensitivity.

Examples of area averaging correction factors along or parallel to main axes *x*, *y* and *z* are presented in Figure 2A. In the *x*-direction, the correction factor is larger at short distances with fall-off close to one at distances larger than 10 mm. In the *y*- and *z*-profiles the averaging effect varies from underestimation at short distances from *x*-axis to overestimation at larger distances. The area correction factor is more even and closer to one overall in profiles with *x* = 5 mm (not shown). The stepwise changes in the *z*-direction result from the interpolation of the tabulated anisotropy function *F*(*r*,*θ*) in the TG-43U1 protocol.

### Angular sensitivity

The angular sensitivity of the diode is presented in Figure 2B. Differences are small (on average ≤ 2%pt., at maximum 6.5%pt.) between rotational positions around the diode axis, and thus the averaged sensitivity has been used for profile correction. Due to a cylindrical metal filter surrounding the diode effective area to eliminate side scatter, there is an abrupt drop in the sensitivity close to α = ± 90°, and this observation has been used when centering the effective point of the diode detector in *x*-direction in angular sensitivity and gold factor measurements where the reference position cannot be taken from a surface.

### Gold correction

**Figure 3:**
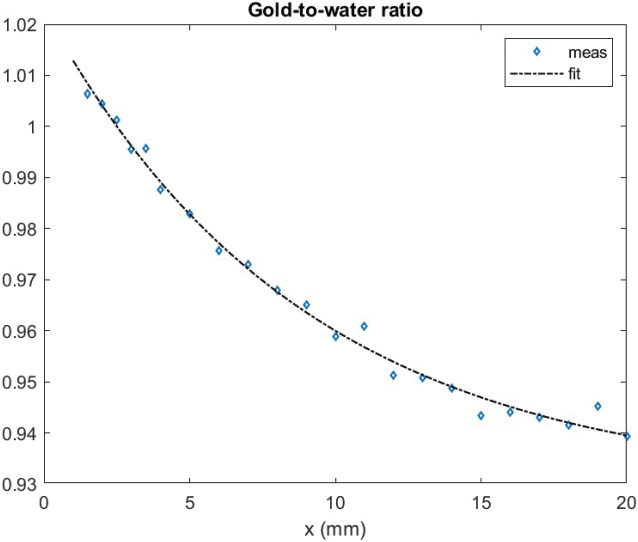
Measured gold-to-water ratio and experimental fit used to correct calculation.

The gold-to-water ratio in *x*-direction from the center of the seed is presented in Figure 3. The measured charge values were not corrected for area averaging or angular sensitivity before division since those are equal for both measurements with and without backing. At distances larger than *x* = 20 mm, the ratio reaches a plateau *C_gold_*(*x*) ≈ 0.94 (not shown).

### Dose profiles and gamma index analysis

The calibration coefficient of the diode is *k*_calib_ = 3.13 ± 0.06 mGy · nC^–1^ (mean ± SD of 10 measurements).

**Figure 4:**
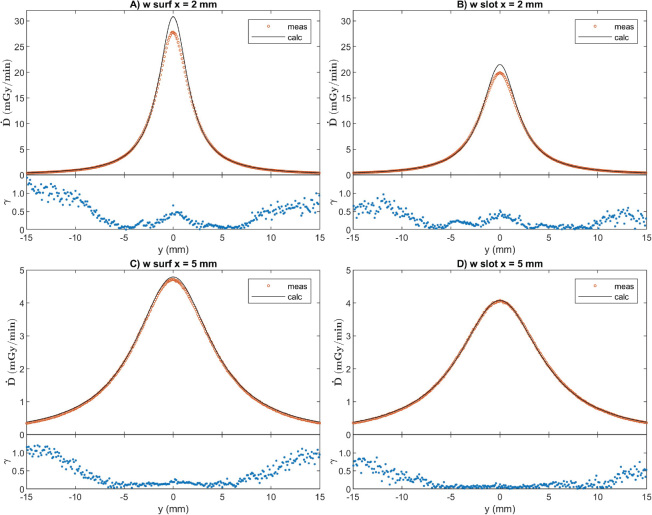
Measured and calculated 1D dose y-profiles with PMMA backing and corresponding γ-values of 1D measurement/3D calculation comparison at 0.3 mm/3% criteria. Abbreviations in figure sections A–D as in Table 1.

**Figure 5:**
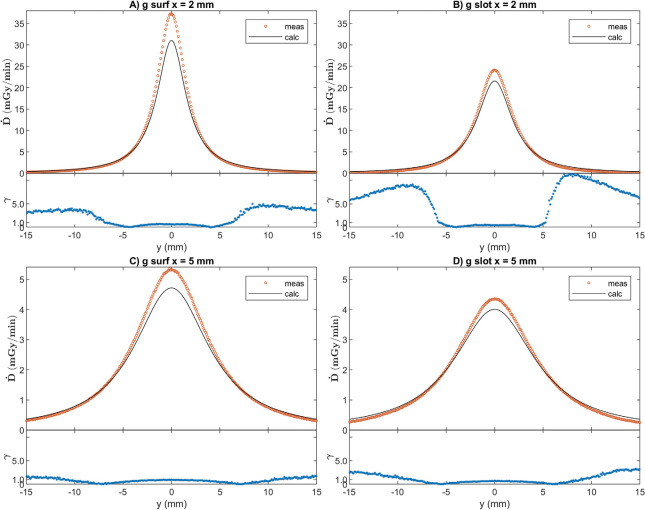
Measured and gold-to-water ratio corrected calculated 1D dose y-profiles with gold backing and corresponding γ-values of 1D measurement/3D calculation comparison at 0.3 mm/3% criteria. Abbreviations in figure sections A–D as in Table 1.

Examples of dose profiles and corresponding γ-values are presented in Figure 4 for measurements with a seed on PMMA-backing and in Figure 5 for a seed on the gold slab. The results of gamma index comparison are presented in Table 1. Profiles were also compared at shorter ranges than the whole measured distance range, and the pass rates are better closer to the *x*-axis and farther from the surface. The calculated dose results with gold backing have overall worse agreement with measurements than those with PMMA backing.

**Table 1:** The gamma indices, that is, the portion of the points that pass the gamma analysis γ ≤ 1, of the measured dose profiles between indicated distance ranges: for xprof from origo and for yprof and yprof from x-axis.

		0.2 mm/2%					0.3 mm/3%					0.5 mm/5%				
		± 5 mm	± 10 mm	± 15 mm	± 20 mm	± 25 mm	± 5 mm	± 10 mm	± 15 mm	± 20 mm	± 25 mm	± 5 mm	± 10 mm	± 15 mm	± 20 mm	± 25 mm
**w**	xprof	**95**	**98**	**96**	81	68	**100**	**100**	**100**	**100**	**100**	**100**	**100**	**100**	**100**	**100**
**surf**	yprof 2 mm	**99**	**93**	74	-	-	**100**	**99**	**90**	-	-	**100**	**100**	**100**	-	-
	zprof 2 mm	**100**	**90**	72	-	-	**100**	**97**	83	-	-	**100**	**100**	**100**	-	-
	yprof 5 mm	**100**	**99**	72	-	-	**100**	**100**	**91**	-	-	**100**	**100**	**100**	-	-
	zprof 5 mm	**100**	**93**	63	-	-	**100**	**100**	79	-	-	**100**	**100**	**100**	-	-
**g**	xprof	*16*	*7*	*32*	50	61	86	**94**	**96**	**97**	**98**	**100**	**100**	**100**	**100**	**100**
**surf**	yprof 2 mm	85	50	*33*	-	-	**100**	64	*43*	-	-	**100**	76	51	-	-
	zprof 2 mm	72	*38*	*26*	-	-	**100**	55	*37*	-	-	**100**	63	*42*	-	-
	yprof 5 mm	*20*	58	*39*	-	-	**100**	**100**	72	-	-	**100**	**100**	**98**	-	-
	zprof 5 mm	*22*	*40*	*26*	-	-	**100**	86	57	-	-	**100**	**98**	73	-	-
**w**	xprof	**95**	**98**	**99**	**99**	**99**	**100**	**100**	**100**	**100**	**100**	**100**	**100**	**100**	**100**	**100**
**slot**	yprof 2 mm	**100**	**100**	**95**	-	-	**100**	**100**	**100**	-	-	**100**	**100**	**100**	-	-
	zprof 2 mm	**99**	**99**	**92**	-	-	**100**	**100**	**100**	-	-	**100**	**100**	**100**	-	-
	yprof 5 mm	**100**	**100**	**94**	-	-	**100**	**100**	**100**	-	-	**100**	**100**	**100**	-	-
	zprof 5 mm	**100**	**100**	**95**	-	-	**100**	**100**	**100**	-	-	**100**	**100**	**100**	-	-
**g**	xprof	61	75	84	86	84	**100**	**100**	**100**	**100**	**100**	**100**	**100**	**100**	**100**	**100**
**slot**	yprof 2 mm	**99**	51	*34*	-	-	**100**	54	*36*	-	-	**100**	60	*40*	-	-
	zprof 2 mm	**100**	55	*37*	-	-	**100**	60	*40*	-	-	**100**	69	*46*	-	-
	yprof 5 mm	80	68	*45*	-	-	**100**	88	59	-	-	**100**	**100**	**72**	-	-
	zprof 5 mm	73	61	*41*	-	-	**100**	82	55	-	-	**100**	**98**	66	-	-

“w” denotes PMMA and “g” gold slab backing, “surf” the surface of the backing and “slot” the 0.4 mm deep (radius of the seed) slot in the backing, “xprof” the depth dose and “yprof” and “zprof” short-axis and long-axis profiles at the denoted distance above the backing. The bolded gamma index values pass the analysis at values over 90% and the values in italics are below 50%.

## Discussion and conclusion

After applying computational correction for the effective detector size (Eq. 1) and experimentally determined correction for detector angular sensitivity (Eq. 2), we have reached a good accuracy for measuring the dose distribution around a single ^125^I seed. When calculated values from TG-43 formalism were used as a reference in water equivalent environment, we reached the pass rate better than 97% of tested points with gamma index 0.3 mm/3% and 93% with 0.2 mm/2%, respectively, within the distance range between 1 mm ≤ *x* ≤ 15 mm and -10 mm ≤ *y, z* ≤ 10 mm. The gamma index analysis of measurement results in water equivalent environment with reference to TG-43 calculations serves as an approach of uncertainty analysis for future applications of diode measurements in more complex cases.

The positioning of the detector with respect to the seed is fairly easy straight above the seed by measuring two orthogonal (*y* and *z*) profiles and centering the detector to the peak. The “depth” direction *x* transverse to the seed was a more challenging task and two strategies were used: (1) touching the holder surface and resetting *x* coordinate accordingly with the correction of diode entrance window thickness and (2) measuring ∆*y* = 8 mm shifted *x*-profile sideways down to below the seed level. This profile presents a drop in the middle, which after angular dependence correction transforms into a peak. Comparing positionings 1 and 2 more than five times, we could confirm the thickness of the diode entrance window indirectly and that the positioning accuracy (as mean difference for coordinate *x* between methods 1 / 2) and precision (1 SD of the zero position) better than 0.1 mm was reached in all orthogonal directions.

Our dosimeter, the Sun Nuclear scanning diode, is quite old and not commercially available anymore. Nevertheless, we chose it because of its thin entrance window of only 0.3 mm. It is much thinner than the current diodes on market, with most having closer to 1 mm windows. We could mechanically reach the 1 mm distance from the seed center. With this very short distance the detector size forms a significant source of uncertainty, but the approximative theoretical correction appeared to give proper results.

While the volume correction of diode signal is experimental, the area averaging effect is calculated against the theoretical shape of dose distribution from TG-43. While we have not used a fully independent test to prove this theoretical method, the good agreement between the measurements against TG-43 calculation supports the validity of this assumption when the seeds are surrounded in water equivalent medium. At the distance of 10 mm, the volume correction is close to unity. At distances larger than 2.5 mm the volume correction is less than 5%. According to our results, the agreement also seems to be reasonable for seeds embedded in holes of shallow depths (≤ seed cylindrical radius). With tighter collimation (deeper slots) the TG-43 dose profile shape does not apply anymore. The next step is to compare the diode results with measurements using radiochromic film (Gafchromic™, Ashland Inc., U.S.) having minimal area averaging effect.

The effect of backing materials has been studied before [8, 9, 15] and the value of the gold-to-water comparison measurements in our study was the need to calculate accurate enough reference data to compare with our profile measurements. The shape of the curve transverse to the seed is similar to what has been reported before [8, 9]. One-dimensional path-length corrections based on similar measurements have been used in an ophthalmic treatment planning system [15]. The gamma results are worse for gold backed profiles compared to simple one-dimensional correction as a function of distance from the seed center than in the case of the water equivalent medium. This is likely due to the approximative nature of the applied one-dimensional gold-to-water correction. When the gamma criteria were relaxed to 0.5 mm/5%, the pass rate reached 98% at 5 mm and 60% at 2 mm and for range -10 mm ≤ *y, z* ≤ 10 mm. The correction obviously works in the depth direction in which it was determined but fails laterally. Physical modeling of the gold correction would require three-dimensional modeling of the scatter and fluorescence x-rays in the exact seed and gold plaque geometry.

This work forms a basis for further dosimetric studies with ophthalmic applicators loaded with several ^125^I seeds, part of them embedded in slots for collimation [12]. The correction factors of a single seed as a function of distance and/or direction can be utilized to generate corrections for multiple seed measurements. The correspondence of single seed results should not be interpreted as a validation of clinical dose calculation as a superposition of multiple seed contributions, but the present results validate the measurement methodology. Further measurements with applicators are necessary to evaluate and adjust the performance of dose calculation algorithm used in treatment planning of ophthalmic brachytherapy.

## Data Availability

Principal author provides data upon request.
